# Indoor Recirculating Aquaculture Versus Traditional Ponds: Effects on Muscle Nutrient Profiles, Texture, and Flavour Compounds in Largemouth Bass (*Micropterus salmoides*)

**DOI:** 10.3390/foods14244339

**Published:** 2025-12-17

**Authors:** Di Feng, Rui Feng, Chang Liu, Lingran Wang, Yongjing Li, Meng Zhang, Miao Yu, Hongxia Jiang, Zhigang Qiao, Lei Wang

**Affiliations:** 1 College of Fisheries, Engineering Technology Research Center of Henan Province for Aquatic Animal Cultivation, Engineering Lab of Henan Province for Aquatic Animal Disease Control, Henan Normal University, Xinxiang 453007, China; 2 Observation and Research Station on Water Ecosystem in Danjiangkou Reservoir, Henan Normal University, Nanyang 474450, China

**Keywords:** simplified F-RAS, muscle texture, amino acids, fatty acids, volatile compounds

## Abstract

This study compared the muscle quality of largemouth bass (*Micropterus salmoides*) reared in a simplified indoor factory-scale recirculating aquaculture system (F-RAS) with those reared in a traditional pond (TP). Juveniles from the same cohort (with a mean initial body mass of approximately 16 g) were stocked into the two systems and reared for ten months. The F-RAS is a high-density indoor system utilising octagonal concrete tanks with an effective water volume of 100 m^3^ and a stocking density of 130 fish m^−3^. The TP is a low-density system, where fish are reared in earthen ponds with a total area of 4000 m^2^ at a density of 1.7 fish m^−3^. At the end of the experiment, 20 fish per group were randomly sampled for morphological analysis, while subsets of 6 fish per group were used for texture analysis, 3 fish per group for water-holding capacity, 3 fish per group for proximate composition, and 9 fish per group (pooled into 3 biological replicates) for amino acid, fatty acid and volatile compound analyses. The results showed that the F-RAS group exhibited superior texture, with significantly higher chewiness, springiness and muscle fibre density. Nutritionally, the F-RAS group had significantly greater crude protein, EPA, DHA and total n-3 fatty acid contents. Although glutamate and leucine levels were lower in the F-RAS group, cysteine and histidine levels were higher. Analysis of volatile compounds indicated improved flavour in the F-RAS group, with a marked reduction in off-flavour compounds such as 1-octen-3-ol and hexanal. Overall, largemouth bass produced in F-RAS showed better muscle quality than those from the TP in terms of texture, nutritional value and flavour. This study provides a reference for future research on the regulation of muscle quality in largemouth bass using a simplified F-RAS.

## 1. Introduction

With the continued growth of the global population, ensuring a sufficient, safe, and nutritious food supply remains an urgent and persistent challenge. Protein, particularly that of animal origin, plays a vital role in human nutrition. However, traditional terrestrial livestock production is under mounting pressure due to constraints on land availability, freshwater resources, and environmental sustainability [1]. Against this backdrop, aquaculture has increasingly emerged as an essential component of global food and nutrition security, offering a sustainable source of high-quality animal protein. Fish and other aquatic foods are among the most nutrient-rich animal-sourced foods worldwide, providing approximately 15% to 16% of global animal protein intake and serving as a key dietary source of essential micronutrients and n-3 fatty acids [2]. Nevertheless, as production volumes expand, aquaculture is subject to increasingly stringent market quality standards and ecological sustainability requirements. Traditional pond (TP) culture still dominates the freshwater sector thanks to its low cost and low technological barriers to entry, and because its intact food web supplies abundant natural feeds that can enhance the availability of amino acids and fatty acids [3]. However, organic matter readily accumulates in these systems, causing water quality fluctuations and eutrophication that can compromise fish health and potentially reduce product safety, nutritional value and flavour attributes [4,5].

Driven by the need for greener, low-carbon and efficient production, recirculating aquaculture systems (RASs) have attracted considerable attention as an intensive farming model. Through physical filtration, biological purification and disinfection, an RAS enables closed-loop water circulation, markedly reducing effluent discharge, improving the stability of the rearing environment and enhancing control over culture conditions [6,7,8]. In comparison with a TP, however, an RAS is also characterised by high initial investment, substantial energy consumption and demanding requirements for technical operation and management [9]. Even so, an RAS can be deployed indoors, and its capacity for precise environmental regulation allows it to buffer aquaculture production against adverse effects of extreme weather events and seasonal variability, providing clear advantages in terms of climate resilience [10]. In addition, several studies indicate that RASs may improve muscle quality and physiological metabolism by promoting moderate swimming activity. For instance, in common carp (*Cyprinus carpio*), the RAS has been shown to markedly enhance growth performance, increase muscle fibre density and improve textural attributes, while also strengthening flavour characteristics [11]. In grass carp (*Ctenopharyngodon idellus*), the RAS improves muscle physical properties and overall health status. Overall, fish reared in RASs generally display superior muscle quality compared with pond-reared counterparts, particularly with respect to physical traits and nutritional composition [12].

Among freshwater fishes, largemouth bass (*Micropterus salmoides*) is an economically important species in North America, South America and several Asian countries. Owing to its rapid growth, delicate flesh and strong environmental adaptability, it has become increasingly popular in the Chinese market and among consumers [13,14]. Previous studies have demonstrated that largemouth bass reared in RASs generally exhibit better muscle quality than those from TP, which makes this species a valuable model for research on improving food quality [15,16]. Most of these investigations, however, have been carried out in fully equipped, highly controllable standard RAS units, whereas systematic studies on simplified RAS configurations with lower resource requirements remain scarce [17,18]. The simplified indoor factory-based recirculating aquaculture system (F-RAS) used in this study was established by converting a disused piggery. The system primarily comprises culture tanks, a side-discharge tank, a vertical-flow sedimentation tank, a microfiltration unit, water pumps, a brush tank and a biofilter tank. Key environmental parameters such as water temperature and aeration are centrally regulated via a control panel. Compared with typical engineered commercial RASs, the system used in this study was not equipped with standardised water treatment units such as ultraviolet disinfection, but retained basic water treatment processes including sedimentation, mechanical filtration, and biofiltration, sufficient to maintain a relatively stable aquaculture environment and continuous water flow, thus constituting a simplified F-RAS [18].

Nevertheless, there is still limited systematic evidence from direct comparisons of muscle quality and flavour profile in largemouth bass between F-RAS and TP culture systems. This study was undertaken using largemouth bass as the model species to compare morphological characteristics, muscle physical properties, nutritional composition, and volatile flavour compounds under the two aquaculture systems. The aim was to determine whether such a simplified F-RAS can achieve quantifiable improvements in muscle quality and flavour, and to provide a scientific basis for evaluating the applicability of the simplified F-RAS and its role in quality regulation.

## 2. Materials and Methods

### 2.1. Experimental Materials

The primary objective of this study was to systematically compare the integrated effects of two aquaculture systems (F-RAS and TP) on the muscle quality of largemouth bass under practical production conditions. Accordingly, the experimental unit was defined as the production system rather than individual ponds or tanks. One F-RAS and one TP, both operated stably for an extended period, were therefore selected as representative prototypes of their respective system types.

The same batch of seedlings was used throughout the experimental process. Largemouth bass fries were procured from Yusong Breeding Family Farm in Dechang County, Sichuan Province, in June 2022 and were subsequently transported to the recirculating water aquaculture facilities of Jiyuan Hongji Sturgeon and Giant Salamander Breeding Co., Ltd. (Jiyuan, China) for rearing. Once the fish reached approximately 4 months of age and an average body mass of 16 g, they were randomly divided into two experimental groups: the TP group and the F-RAS group.

The F-RAS group was cultured in an indoor factory-based recirculating aquaculture system reconstructed from an abandoned pigsty, with a rearing density of 130 fish per cubic metre. The culture environment was maintained under controlled conditions, with ammonia nitrogen levels ranging from 0.05 to 0.11 mg/L, nitrite concentrations from 0.15 to 0.2 mg/L, pH values between 6.9 and 7.7, and dissolved oxygen levels maintained in the range of 6.3 to 7.9 mg/L. The fish were raised in octagonal concrete tanks, each with a surface area of 60 m^2^ and a depth of 1.7 m, providing an effective culture volume of approximately 100 m^3^ per tank. Throughout this period, water quality parameters were monitored daily, and a partial water change was performed whenever any parameter approached the upper limit of its target range.

The TP group was cultured in traditional earthen ponds, with a stocking density set at 1.7 fish per cubic metre. The total pond area covered approximately 4000 m^2^ with an average water depth of 2.2 m. Groundwater was utilised as the water source, with ammonia nitrogen levels fluctuating between 0.12 and 0.3 mg/L, nitrite levels from 0.35 to 0.55 mg/L, pH values ranging from 7.8 to 8.5, and dissolved oxygen concentrations maintained between 4.0 and 6.2 mg/L.

During the experimental period, both groups were uniformly fed with Huifu feed (Huifu Feed, Jiaxing, China). After 10 months of separate culture under the respective systems, 20 largemouth bass were randomly selected from each group for further analysis. To minimise the influence of individual variation within each system on the experimental results, appropriate biological replication and sample pooling strategies were implemented at the tissue level. The number of samples for each experimental testing indicator was determined based on the biological variability of the indicator itself, the sensitivity of the analytical technique, and reference to relevant studies [12,15]. All tissue samples were consistently collected from the same anatomical location, and the analytical procedures were strictly standardised, thereby ensuring that the data from each group reliably and accurately reflected the average muscle quality under the corresponding culture model. The specific number of biological replicates and pooling strategies for each parameter are detailed in the respective methodological subsections.

### 2.2. Schematic Diagram of F-RAS

**Figure 1:**
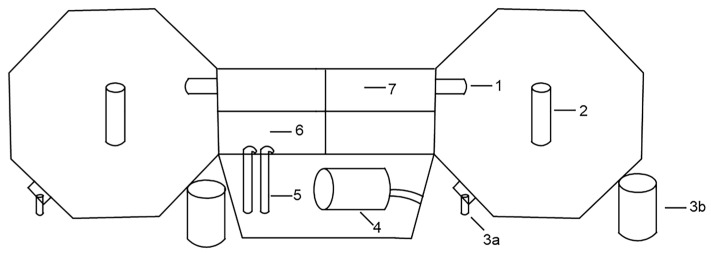
Schematic diagram of the F-RAS. 1: Inlet; 2: Isolation net; 3a: Side discharge tank; 3b: Vertical flow sedimentation tank; 4: Microfiltration unit; 5: Water pump; 6: Brush tank; 7: Biofilter tank.

The operational flow of the F-RAS is illustrated in Figure 1. Recirculating water is driven by two water pumps, each with a rated power of 800 W, delivering a total flow rate of approximately 100 m^3^ per hour. The water is first directed through a brush tank and a biofilter tank for initial filtration before entering the culture tank via the inlet. A centrally positioned isolation net within the culture tank serves to guide the water flow and separate the fish stock. The water then passes sequentially through the side discharge tank and the vertical flow sedimentation tank, where solid particles are removed by gravity settling, before entering the microfiltration unit for final fine filtration. The treated water is then returned to the culture tank, completing a closed-loop cycle. The system is arranged symmetrically on both sides to enhance hydraulic circulation and structural balance. Each filtration and return module operates primarily via gravitational force and mechanical brushing, forming a closed water treatment pathway dominated by physical purification processes.

### 2.3. Morphological Characteristics of Largemouth Bass

Fish were anaesthetised with 200 mg L^−1^ MS-222 (NaHCO_3_-buffered), euthanised in 1000 mg L^−1^ MS-222 until complete and irreversible cessation of opercular movement, and immediately subjected to cervical dislocation. Subsequently, 20 largemouth bass were randomly sampled from each of the TP and F-RAS groups to measure the body length and final body weight, with condition factor calculated according to the following formula:

(1) CF = 100 × (FBW/BL^3^)

where CF is the condition factor (dimensionless), FBW is the final body weight (g), and BL is body length (cm).

### 2.4. Physical Properties of Dorsal Muscle

For the determination of water-holding capacity (WHC), the experimental procedures and final calculation formulae were based on the method described in [19], with appropriate modifications to accommodate the experimental materials. For each group, 5 g of white muscle was taken from the dorsal region of each fish as experimental material. A total of 3 fish were used per group, and six parameters were sequentially measured: centrifugal loss, stored loss, cooked rate, frozen leakage, liquid loss, and drip loss.

For textural characterisation, cubes of approximately 1.0 cm were cut from the experimental material obtained from each fish in each group. A total of 6 fish were used per group. The texture properties, including hardness, adhesiveness, chewiness, springiness, cohesiveness, shearing, and resilience, were analysed using a TA-XT plus texture analyser. Cooked muscle samples were boiled for 5 min, cooled, and analysed under the same conditions as raw muscle samples.

Approximately 0.5 cm cubes were cut from the experimental material obtained from each fish in each group. A total of 3 fish were used per group. The tissue samples were subsequently fixed by complete immersion in 4% tissue cell fixative (P1110, Solarbio, Corp., Beijing, China). Thereafter, dehydration, wax infiltration, embedding, sectioning, and staining were performed sequentially. Following processing, the structure, diameter, and density of muscle fibres were analysed in detail using CaseViewer software 2.4.

### 2.5. Nutritional Composition of Dorsal Muscle

The experimental methods and reference standards for the conventional nutrition (moisture, ash, crude fat, crude protein), amino acids, and fatty acids involved in this study are based on the description provided in [15]. For each group, a total of three fish were used for the analysis of conventional nutrition, and nine fish randomly pooled into 3 biological replicates were used for the determination of amino acids and fatty acids.

### 2.6. Volatile Compound Analysis

Volatile compounds in muscle samples were identified and analysed using a FlavourSpec^®^ flavour analyser (G.A.S., Dortmund, Germany), following the methodology described in [20]. For each group, nine fish were randomly selected and pooled into 3 biological replicates for analysis.

### 2.7. Statistical Analysis

All statistical analyses were conducted using SPSS version 26.0 (IBM Corp., Armonk, NY, USA). All variables were assessed as continuous and normally distributed prior to analysis to ensure compliance with the assumptions of parametric testing. Independent samples t-tests were employed to assess differences between the TP and F-RAS groups, with a predefined significance level set at *α* = 0.05. Results with *p*-values between 0.01 and 0.05 were considered statistically significant, whereas values below 0.01 were regarded as highly significant. Experimental data are expressed as means ± standard error of the mean (SEM), with n denoting the number of biological replicates.

## 3. Results

### 3.1. Morphological Characteristics

As shown in Table 1, all morphometric parameters of largemouth bass showed no significant differences between the two groups (*p* > 0.05), as visually confirmed by the representative photographs in Supplementary Figure S1.

### 3.2. WHC

Quantitative analysis of muscle WHC demonstrated no intergroup divergence across six key parameters (*p* > 0.05), with detailed comparative metrics systematically tabulated in Table S1.

### 3.3. Textural Characteristics

**Figure 2:**
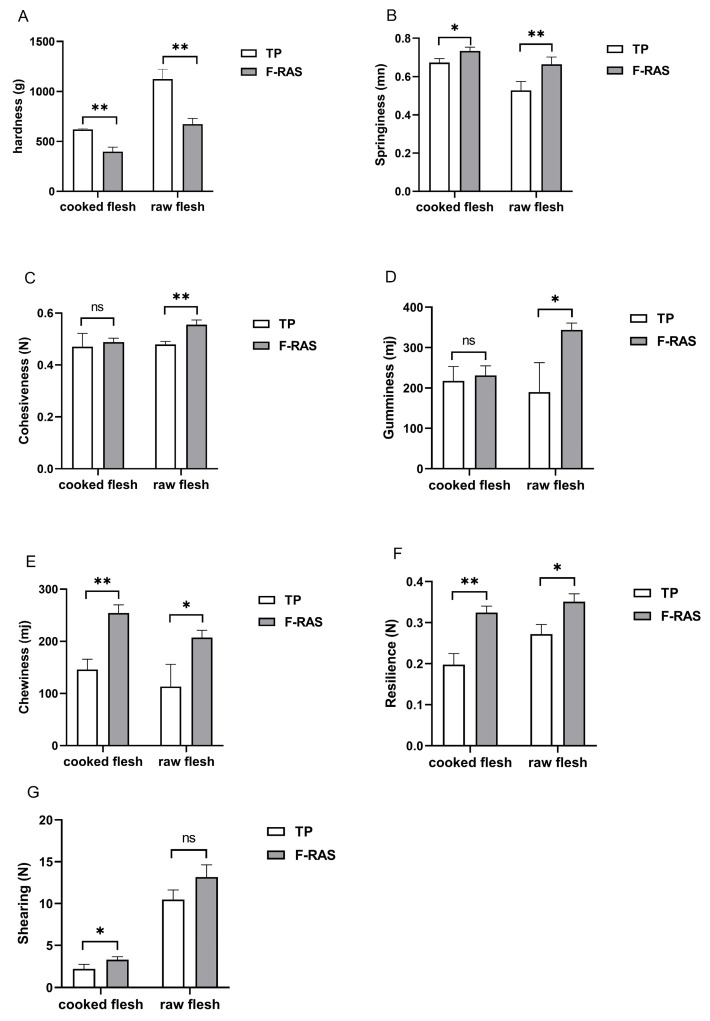
Textural characteristics of raw and cooked largemouth bass muscle from both groups (*n* = 6 per group). * indicates a significant difference (*p* < 0.05); ** indicates a highly significant difference (*p* < 0.01); ns indicates no significant difference (*p* > 0.05). Raw-fillet and cooked-fillet texture of largemouth bass muscle cultured under two aquaculture modes: (**A**) Hardness; (**B**) Springiness; (**C**) Cohesiveness; (**D**) Gumminess; (**E**) Chewiness; (**F**) Resilience; (**G**) Shearing.

The textural characteristics of largemouth bass muscle in the two aquaculture systems are shown in Figure 2. The results comparing muscle samples from both groups under two different conditions are as follows: In raw meat, except for shearing and hardness, largemouth bass muscle in the F-RAS group was significantly higher in chewiness, gumminess, and resilience (*p* < 0.05) and highly significant in springiness and cohesiveness (*p* < 0.01). In cooked meat, except for cohesiveness, gumminess, and hardness, largemouth bass muscle in the F-RAS group was significantly higher in springiness and shearing (*p* < 0.05) and highly significant in chewiness and resilience (*p* < 0.01).

### 3.4. Muscle Fibre Characteristics

**Figure 3:**
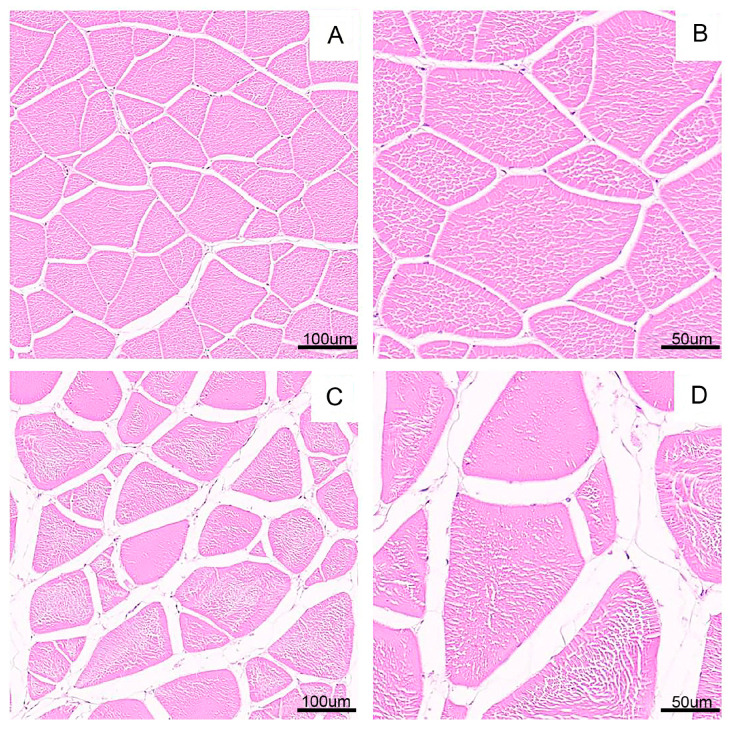
Muscle cross-section of largemouth bass muscle from both groups. (**A**,**B**) 100 µm, 50 µm in F-RAS group; (**C**,**D**) 100 µm, 50 µm in TP group.

As can be seen from Table 2, in contrast to the TP group, the long and short diameters of the largemouth bass muscle fibres in the F-RAS group were significantly reduced (*p* < 0.01), while the fibre density was markedly increased (*p* < 0.01). The cross-sectional images of the muscle fibres are shown in Figure 3.

### 3.5. Muscle Nutrient Composition

Compared with the F-RAS group, significantly reduced concentrations of crude protein were detected in the TP group (*p* < 0.05), as shown in Table 3.

In terms of fatty acid composition, the F-RAS group exhibited a highly significantly lower content of monounsaturated fatty acids (ΣMUFA) compared with the TP group (*p* < 0.01), while the content of polyunsaturated fatty acids (ΣPUFA) was significantly higher (*p* < 0.05). Furthermore, the total n-6 fatty acids (Σn-6) content in the F-RAS group was significantly lower than that in the TP group (*p* < 0.05), whereas its total n-3 fatty acids (Σn-3) content was highly significantly higher (*p* < 0.01). In addition, the levels of EPA (C20:5n-3) and DHA (C22:6n-3) in the muscle of the F-RAS group were also highly significantly greater than those in the TP group (*p* < 0.01), as shown in Table 4.

Regarding amino acid composition, the TP group exhibited significantly higher glutamic acid, proline, and leucine contents, but significantly lower cysteine and histidine contents, relative to the F-RAS group (*p* < 0.05). However, despite these differences, the essential amino acids (EAAs), umami amino acids (UAAs), and nonessential amino acids (NEAA) levels remained statistically similar between the two groups (*p* > 0.05), as shown in Table 5.

### 3.6. Comparative Analysis of Muscle Principal Components

**Figure 4:**
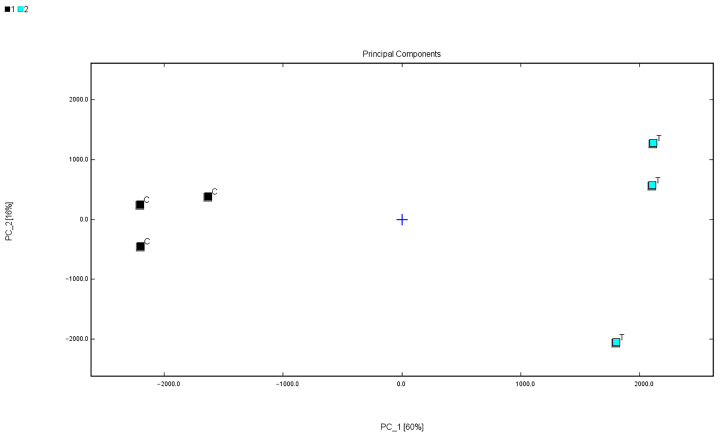
PCA score plot of volatile components in largemouth bass muscle. (C) F-RAS group; (T) TP group; PC1 and PC2 represent the first and second principal components, explaining 60% and 16% of the total variance, respectively.

As shown in Figure 4, the PCA (Principal Component Analysis) results clearly separate the muscle samples from the two rearing systems into distinct groups. This clustering indicates that the overall composition of volatile flavour compounds differs noticeably between the two groups. In other words, the two aquaculture environments led to distinct flavour profiles in the fish muscle, and PCA effectively highlights this variation by reducing complex data into visual patterns.

### 3.7. Fingerprinting and Qualitative Analysis of Muscle Composition

**Figure 5:**
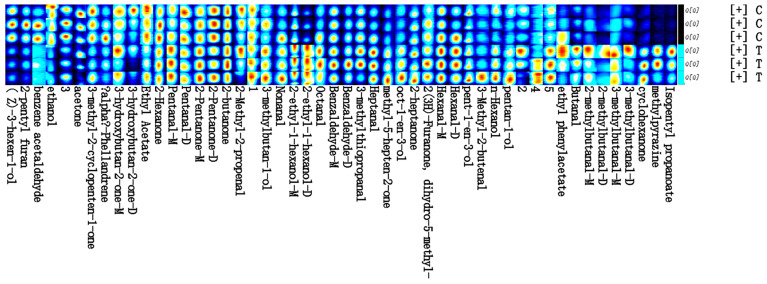
Fingerprinting of muscle composition in largemouth bass. (C) F-RAS group; (T) TP group.

Based on the significance of the difference between the two groups of compounds detected, relative peak area, mass spectrometry match, and other dimensions, the characteristic peaks were screened to further analyse and determine the differences in the flavour substances between the groups. Based on these characteristic peaks, a fingerprint spectrum was constructed in the form of a heat map, as shown in Figure 5. The depth of the colour and the flavour compounds is proportional to the relationship between the colour depth and the flavour compounds, which horizontally reflects the similarities and differences in flavour compounds in and between the groups, and vertically can clearly compare the differences in concentration of each characteristic compound between groups. We can clearly compare the concentration differences of the characteristic compounds between the groups, and then qualitative analysis of these compounds was conducted. A total of 50 flavour compounds were characterised, but due to the lack of complete information in the database, only 45 were matched by the database, of which 5 in the fingerprints were marked with Arabic numerals to represent the unidentified compounds that were not matched by the database, and 16 compounds in the research object were identified based on the change in concentration conditions. In addition, 16 compounds in the study population were retained in the form of monomers, where a single molecule exists independently, and dimers, where two molecules are bound by intermolecular forces, based on the change in concentration conditions, and were distinguished by -M and -D, respectively. The complete information on these flavour compounds is provided in Supplementary Table S2.

The data were obtained by independent samples *t*-test, and when comparing with the TP group, a statistically significant increase was observed for phenylacetaldehyde, 2-pentanone-D, and ethanol in the F-RAS group, and the opposite was true for methyl-5-hepten-2-one, 2-pentanone-M, 2-methylbutyraldehyde-M, and 3-methyl-1-butanol (*p* < 0.05). In addition, the content of (Z)-3-hexen-1-ol was highly significantly increased, whereas 2-ethylhexanol-M, 2-ethylhexanol-D, heptanal, isoamyl propionate, 1-octen-3-ol, cyclohexanone, pentanal-M, 3-methylbutanal-M, butanal, methylpyrazine, and nonanal were significantly decreased (*p* < 0.01). These findings indicate that muscle flavour undergoes different changes due to the different rearing methods.

## 4. Discussion

### 4.1. Effects of Different Aquaculture Systems on Physical Properties of Largemouth Bass Muscle

In terms of physical characteristics, the F-RAS group exhibited smaller fibre diameters and short diameters, along with a greater muscle fibre density, which may be attributed to the specific culture environment of the RAS. In such systems, higher stocking densities and the continuous presence of water currents are likely to induce sustained and spontaneous swimming behaviour [21]. The resulting prolonged mechanical stimulation can influence muscle development by promoting the expression of myogenic regulatory factors, thereby triggering the differentiation of satellite cells and the formation of new muscle fibres [15]. These changes are likely to induce microstructural adaptations in muscle tissue, characterised by a reduced fibre diameter and a more densely packed arrangement [22].

It is worth noting that muscle fibre density is closely associated with the eating quality of fish flesh. A densely arranged fibre structure may enhance the WHC of muscle cells, thereby improving muscle texture to a certain extent and increasing firmness and springiness. These findings are consistent with the significantly higher values observed for multiple textural parameters in the F-RAS group compared to the TP group [23]. Furthermore, regular and moderate exercise has also been shown to significantly enhance muscle fibre compactness, thereby improving overall textural quality and increasing the springiness and chewiness of fish flesh [24]. These findings suggest that the rearing conditions of the F-RAS may have provided the fish with an appropriate level of exercise intensity, thereby facilitating structural optimisation and functional enhancement of muscle tissue. Meanwhile, firmer texture and denser muscle fibre structure are generally associated with better shelf-life quality and higher consumer acceptance, indicating that F-RAS-cultured fish may possess stronger commercial competitiveness in the fresh-sale market [25].

### 4.2. Effects of Different Aquaculture Systems on Nutrient Composition of Largemouth Bass Muscle

The nutritional composition of muscle serves as a key indicator for assessing the quality and nutritional value of fish fillets, and its content is commonly influenced by environmental factors such as culture methods and stocking density. Previous studies have shown that continuous water flow in recirculating aquaculture systems forces fish to maintain sustained swimming activity. In species such as grass carp and yellow catfish (*Pelteobagrus fulvidraco*), this persistent exercise stimulus has been demonstrated to effectively enhance muscle protein deposition [26,27]. In the present study, the higher crude protein content observed in the F-RAS group suggests that the continuous hydraulic environment provided by this system similarly induces exercise-related metabolic stimulation in largemouth bass, thereby improving the efficiency of protein deposition in muscle tissue. These findings confirm that the simplified F-RAS can effectively increase the crude protein content in the muscle of largemouth bass.

In the present study, the differences in the muscle amino acid profile of largemouth bass between the two culture systems were primarily reflected in the contents of specific amino acids, rather than in the total levels of essential amino acids or nonessential amino acids. Specifically, the contents of glutamic acid, leucine, and proline were lower, while those of cysteine and histidine were significantly higher in the F-RAS group. These findings are likely attributable to the increased basal activity induced by the continuous water flow in this system. Under such conditions, largemouth bass exhibited higher overall energy expenditure and may have preferentially utilised amino acids such as glutamic acid for energy metabolism, consistent with the known role of amino acids as an energy source in this species [28,29]. Meanwhile, the elevated levels of histidine and cysteine may reflect physiological adaptations to maintain metabolic homeostasis in a flowing-water environment: histidine contributes to pH buffering in muscle [30,31], and cysteine serves as a rate-limiting precursor for glutathione synthesis, thereby enhancing antioxidant defence against exercise-induced oxidative stress [32]. Together, these findings suggest that the continuous water flow in the F-RAS not only promotes protein deposition but may also alter the metabolic partitioning of specific amino acids in muscle. Future studies should further investigate and measure key physiological indicators related to amino acid metabolism and oxidative stress, which would contribute to elucidating the regulatory mechanisms by which water flow affects the nutritional quality of largemouth bass muscle.

Within the context of human dietary patterns, fish are a valuable source of high-quality fatty acids, particularly n-3 fatty acids, which are known to play crucial nutritional roles in early neurological development, visual function, and cardiovascular health [33,34]. Specifically, n-3 fatty acids, such as EPA and DHA, have been widely recognised for their physiological importance in infants and adults alike [2]. The present study revealed that under the F-RAS culture mode, the level of ΣPUFA in fish muscle was significantly increased, with notable elevations in nutritionally important Σn-3, EPA, and DHA levels, while the proportion of ΣMUFA showed a corresponding decrease. This fatty acid profile aligns more closely with the lipid nutrition characteristics of a healthy diet, suggesting that the F-RAS can optimise the nutritional structure of fatty acids in largemouth bass muscle.

Notably, previous studies have reported differential effects of exercise intensity on the fatty acid composition of fish muscle: low- to moderate-intensity exercise generally promotes the synthesis and deposition of EPA and DHA in juvenile qingbo (*Spinibarbus sinensis*), whereas high-intensity exercise may adversely affect the accumulation of these two fatty acids in the muscle of both rainbow trout (*Oncorhynchus mykiss*) and juvenile qingbo [22,35]. Under the conditions of the present study, the F-RAS group exhibited favourable trends in certain muscle fatty acid composition parameters, particularly with significantly increased levels of Σn-3, EPA, and DHA. This indicates a shift in the overall fatty acid profile towards a composition more beneficial to human health, demonstrating improved nutritional characteristics.

Since the water flow velocity was not measured in this study, it is necessary for future research to systematically evaluate the effects of different water flow intensities on the muscle quality of largemouth bass within the F-RAS platform. This can be achieved by establishing specific gradients of water flow velocity, aiming to identify the key parameter ranges that not only promote healthy fish growth but also directionally improve muscle quality.

### 4.3. Effects of Different Aquaculture Systems on Volatile Compounds in Largemouth Bass Muscle

Volatile flavour compounds are a key component in evaluating the flavour characteristics of fish muscle, and variations in their types and relative abundances can markedly influence the sensory quality of aquatic products as well as consumer acceptance [36]. In the present study, a total of 41 volatile compounds were identified, mainly comprising aldehydes, alcohols, ketones, and esters. Among these, aldehydes made a relatively greater contribution to the overall flavour perception due to their generally lower odour thresholds, with straight-chain aldehydes derived predominantly from lipid oxidation being particularly representative [37]. For instance, hexanal and heptanal are recognised as important products formed during the oxidation of PUFA, such as linoleic acid. These compounds are typically associated with flavour descriptors such as “fishy” or “stale” and hold distinctive significance in the flavour profiles of freshwater fish [38].

In addition, branched-chain aldehydes such as 3-methylbutanal and 2-methylbutanal are primarily derived from the Strecker degradation of branched-chain amino acids, including isoleucine and valine, during storage and protein breakdown. These compounds are considered to be potentially associated with the formation of off-flavours such as fermented, metallic, or otherwise unpleasant odours [39]. The elevated concentrations of 2-methylbutanal-M and 3-methylbutanal-M in the muscle of the TP group observed in this study suggest that fish reared under TP conditions may be more susceptible to the accumulation of branched-chain aldehydes linked to unpleasant odours during storage or tissue degradation, potentially exerting a negative impact on the overall sensory quality of the final product.

Apart from aldehydes, other types of volatile compounds also play important roles in flavour development. Among alcohols, 1-octen-3-ol is characterised by a relatively low odour threshold and is commonly associated with distinct earthy and mushroom-like aromas in aquatic products [40,41]. Methylpyrazine, a typical pyrazine derivative, is frequently linked to undesirable sensory attributes such as earthy or musty odours [42]. Regarding ketones, 2-pentanone-D has been suggested to be associated, to a certain extent, with umami perception [43]. Additionally, within the alcohol category, (Z)-3-hexen-1-ol and high concentrations of 2-ethyl-1-hexanol are known to contribute grassy and unpleasant odours, respectively [44,45].The results of this study indicate that the TP group showed more pronounced accumulation of multiple off-flavour-related volatile compounds, whereas the F-RAS group exhibited higher relative abundances of compounds with positive flavour contributions, such as 2-pentanone-D and (Z)-3-hexen-1-ol. These findings suggest that the F-RAS not only suppresses the accumulation of typical off-flavour substances but also promotes the formation of compounds associated with grassy aroma and umami-related notes, demonstrating its positive role in modulating the overall flavour quality of fish muscle.

Although this study did not directly investigate the microbial or enzymatic mechanisms underlying off-flavour formation, previous research has indicated that factors such as organic pollutants, water quality fluctuations, and feed residues commonly present in TP rearing environments may contribute to the accumulation of various sensory-negative volatile compounds in fish muscle. These include compounds associated with fishy, earthy, mushroom-like, grassy, musty, and metallic odours [46]. Such off-flavours typically originate from lipid oxidation and protein degradation, and their co-existence in fish flesh may produce additive sensory effects, thereby intensifying the perception of off-flavours and reducing overall flavour acceptability [38,47]. However, F-RAS, through processes such as recirculating purification and pollutant isolation, may help to reduce the formation of off-flavour compounds. Although the underlying mechanisms remain to be fully elucidated, the current data suggest that the F-RAS rearing model offers a clear advantage in improving the flavour quality of fish muscle.

In future studies, stricter standardisation of sample sizes and increased biological replication are recommended. Although the present experiment was conducted with scientific rigour and yielded reliable results, such improvements would enhance the generalisability and credibility of the findings and provide a stronger basis for exploring the mechanisms by which aquaculture systems influence fish muscle quality.

## 5. Conclusions

This study demonstrates that the muscle fibre structure in the F-RAS group was more compact, characterised by shorter fibre diameters and a higher fibre density, which corresponded with significant improvements in texture parameters such as chewiness and springiness. At the nutritional level, the F-RAS group exhibited notably higher contents of crude protein, Σn-3 fatty acids, EPA, and DHA, while alterations in certain amino acids may reflect metabolic adaptation of the fish to the F-RAS environment. Regarding volatile flavour compounds, the F-RAS group showed improved flavour characteristics, exemplified by a significant reduction in off-flavour compounds such as 1-octen-3-ol and hexanal. Overall, the simplified F-RAS can optimise the physical properties, nutritional quality, and flavour characteristics of largemouth bass muscle, offering a promising technical approach for the production of high-quality largemouth bass.

## Figures and Tables

**Table 1:** Morphometric parameters of largemouth bass muscle from both groups (*n* = 20).

Parameters	TP	F-RAS
IBW (g)	16 ± 0.19	16 ± 0.58
FBW (g)	309.25 ± 20.62	319.15 ± 9.18
BL (cm)	23.13 ± 0.54	23.14 ± 0.25
CF (g/m^3^)	2.44 ± 0.05	2.58 ± 0.06

CF, condition factor; W, body weight (g); BL, body length (cm); FBW, final body weight (g); IBW, initial body weight (g).

**Table 2:** Muscle fibre characteristics of largemouth bass from both groups (*n* = 3).

Aquaculture System	Short Diameter/µm	Long Diameter/µm	Density/(n/mm^2^)
TP	112.53 ± 0.55 **	139.60 ± 2.42 **	126.97 ± 2.18 **
F-RAS	81.03 ± 3.55	115.11 ± 1.48	169.65 ± 8.54

** indicates an extremely significant difference (*p* < 0.01).

**Table 3:** Conventional nutrient composition contents of largemouth bass muscle from both groups (% of wet matter basis, *n* = 3).

Parameters	TP	F-RAS
Moisture	76.70 ± 0.19	77.09 ± 0.78
Ash	1.16 ± 0.01	1.17 ± 0.05
Crude lipid	2.60 ± 0.03	3.02 ± 0.40
Crude protein	18.50 ± 0.06 *	20.04 ± 0.33

* indicates a significant difference (*p* < 0.05).

**Table 4:** Fatty acid contents of largemouth bass muscle from both groups (%, *n* = 3).

Fatty Acids	TP	F-RAS
C10:0	0.49 ± 0.03 **	1.19 ± 0.12
C12:0	0.70 ± 0.04 **	1.61 ± 0.12
C14:0	1.84 ± 0.12 **	2.65 ± 0.10
C15:0	0.26 ± 0.01	0.30 ± 0.05
C16:0	20.42 ± 0.81	18.23 ± 0.08
C18:0	5.64 ± 0.18 *	7.52 ± 0.63
C20:0	0.31 ± 0.01	0.28 ± 0.05
ΣSFA	31.79 ± 0.91	29.67 ± 0.87
C15:1	0.23 ± 0.09	0.53 ± 0.20
C16:1	3.67 ± 0.12 *	3.06 ± 0.24
C18:1n-9c	29.22 ± 0.32 **	24.72 ± 0.20
C20:1	1.00 ± 0.01 *	0.87 ± 0.04
C22:1n-9	0.64 ± 0.01 **	0.90 ± 0.01
C24:1n-9	0.21 ± 0.01 *	0.41 ± 0.05
ΣMUFA	34.98 ± 0.44 **	30.48 ± 0.25
C18:2n-6c	23.97 ± 0.30 **	21.45 ± 0.32
C18:3n-3	2.10 ± 0.05 *	1.69 ± 0.08
C20:2	0.55 ± 0.01 **	0.60 ± 0.01
C20:3n-6	0.59 ± 0.02 **	1.21 ± 0.05
C20:5n-3 (EPA)	0.94 ± 0.02 **	1.48 ± 0.06
C22:6n-3 (DHA)	6.36 ± 0.08 **	10.84 ± 0.16
ΣPUFA	34.50 ± 0.47 *	37.26 ± 0.68
EPA + DHA	7.30 ± 0.10 **	12.31 ± 0.20
Σn-3	9.40 ± 0.15 **	14.01 ± 0.24
Σn-6	24.55 ± 0.33 *	22.66 ± 0.46

ΣSFA, ΣMUFA, ΣPUFA, Σn-3, and Σn-6 denote the total saturated, monounsaturated, polyunsaturated, omega-3, and omega-6 fatty acids, respectively. * indicates a significant difference (*p* < 0.05), and ** indicates an extremely significant difference (*p* < 0.01).

**Table 5:** Amino acid composition contents of largemouth bass muscle from both groups (%, *n* = 3).

Amino Acids	TP	F-RAS
Aspartic acid	10.24 ± 0.04	10.22 ± 0.04
Threonine	4.63 ± 0.04	4.67 ± 0.04
Serine	3.38 ± 0.07	3.57 ± 0.10
Glutamate	16.10 ± 0.05 *	15.83 ± 0.06
Glycine	4.99 ± 0.04	5.14 ± 0.05
Alanine	6.36 ± 0.01	6.38 ± 0.04
Cysteine	1.18 ± 0.01 *	1.22 ± 0.01
Valine	5.23 ± 0.04	5.22 ± 0.02
Methionine	3.27 ± 0.03	3.29 ± 0.01
Isoleucine	5.06 ± 0.03	5.07 ± 0.04
Leucine	8.63 ± 0.03 *	8.51 ± 0.02
Tyrosine	3.50 ± 0.05	3.65 ± 0.03
Phenylalanine	4.77 ± 0.02	4.67 ± 0.04
Lysine	10.26 ± 0.07	10.27 ± 0.07
Histidine	2.34 ± 0.03 *	2.43 ± 0.02
Arginine	6.46 ± 0.05	6.37 ± 0.05
Proline	3.59 ± 0.02 *	3.52 ± 0.02
ΣEAA	50.65 ± 0.04	50.49 ± 0.06
ΣNEAA	11.66 ± 0.10	11.94 ± 0.11
ΣUAA	37.69 ± 0.11	37.57 ± 0.17

ΣEAA, ΣNEAA, and ΣUAA denote the total essential, nonessential, and umami amino acids, respectively. * indicates a significant difference (*p* < 0.05).

## Data Availability

The data supporting this study are provided within the article and Supplementary Materials. Additional information is available from the corresponding authors upon request.

## Supplementary Materials

The following supporting information can be downloaded at: https://www.mdpi.com/article/10.3390/foods14244339/s1, Table S1: WHC of largemouth bass muscle from both groups (%, *n* = 3); Table S2: Qualitative Analysis of Muscle Volatile Components in Largemouth Bass from both groups (%, *n* = 3). Figure S1. Representative photographs of the largemouth bass from both groups. (A) F-RAS group; (B) TP group.

## Abbreviations

The following abbreviations are used in this manuscript:

F-RAS	Factory-based recirculating aquaculture system
TP	Traditional pond
WHC	Water-holding capacity
ΣMUFA	The content of monounsaturated fatty acids
ΣPUFA	The content of polyunsaturated fatty acids
Σn-6	The total n-6 fatty acids
Σn-3	The total n-3 fatty acids
EPA	C20:5n-3
DHA	C22:6n-3
EAA	The total essential amino acids
UAA	The total umami amino acids
NEAA	The total nonessential amino acids
PCA	Principal component analysis
