# Supplementary material for: Indoor Recirculating Aquaculture Versus Traditional Ponds: Effects on Muscle Nutrient Profiles, Texture, and Flavour Compounds in Largemouth Bass (Micropterus salmoides)
Source: Foods. 2025 Dec 17;14(24):4339. doi: 10.3390/foods14244339 (PMC12733158; doi:10.3390/foods14244339)
Supplement: Supplementary file 1 [file foods-14-04339-s001.zip › foods-4020457-supplementary.pdf]

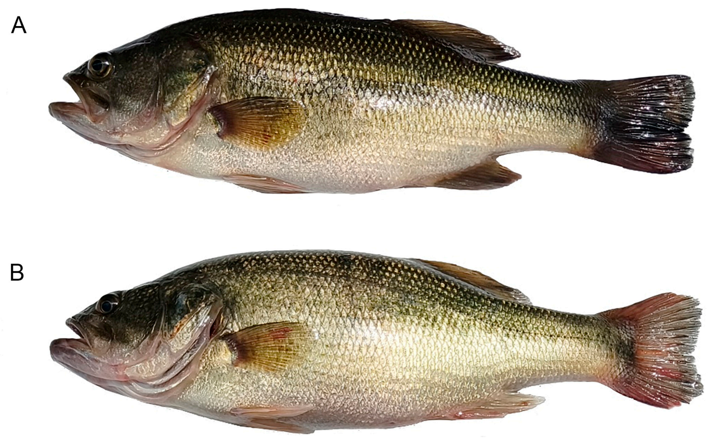

Figure S1. Representative photographs of the largemouth bass from both groups. (A) F-RAS group; (B) TP group.

Table S1. WHC of largemouth bass muscle from both groups (%, n = 3).

Parameters	TP	F-RAS
Centrifugal loss	6.98 ± 1.76	5.30 ± 0.75
Stored loss	2.86 ± 0.38	2.41 ± 0.14

Parameters	TP	F-RAS
Cooked rate	83.69 ± 2.19	85.89 ± 1.97
Frozen leakage	1.92 ± 0.13	2.09 ± 0.18
Liquid loss	21.56 ± 0.57	20.74 ± 0.83
Drip loss	5.47 ± 0.54	5.09 ± 0.25

Table S2. Qualitative Analysis of Muscle Volatile Components in Largemouth Bass from both groups (%, *n* = 3).

Count	Compound	CAS#	RI	Rt	Dt	Peak intensity	Comment

			Formula	MW		[sec]	[a.u.]			
								TP	F-RAS	
1	ethyl phenylacetate	C101973	$C_{10}H_{12}O_2$	164.2	1238.3	1149.267	1.28943	270.11 ± 40.56	226.36 ± 57.00	
2	Nonanal	C124196	$C_9H_{18}O$	142.2	1101.2	774.122	1.4776	339.06 ± 10.05	288.90 ± 42.53	
3	2-ethyl-1-hexanol-M	C104767	$C_8H_{18}O$	130.2	1040.8	650.418	1.41631	4970.88 ± 184.63**	2949.56 ± 84.26	Monomer
4	2-ethyl-1-hexanol-D	C104767	$C_8H_{18}O$	130.2	1039.8	648.7	1.80264	3576.76 ± 213.81**	812.11 ± 46.55	Dimer
5	Octanal	C124130	$C_8H_{16}O$	128.2	1001.3	580.548	1.40516	384.01 ± 31.24	261.43 ± 35.15	
6	3-methyl-2-cyclopenten-1-one	C2758181	C_6H_8O	96.1	972.2	519.842	1.10613	143.86 ± 15.62	186.15 ± 3.76	
7	Benzaldehyde-M	C100527	C_7H_6O	106.1	956.7	488.916	1.1507	1195.03 ± 85.87	1027.82 ± 30.75	Monomer
8	Benzaldehyde-D	C100527	C_7H_6O	106.1	956.7	488.916	1.46646	394.85 ± 54.30	296.21 ± 16.35	Dimer

9	3-methylthiopropanal	C3268493	C_4H_8OS	104.2	902.5	394.304	1.08985	185.06 ± 37.01	167.33 ± 5.78
10	Heptanal	C111717	$C_7H_{14}O$	114.2	896.3	384.874	1.33453	454.43 ± 8.55**	297.40 ± 30.81
11	benzene acetaldehyde	C122781	C_8H_8O	120.2	1048	664.099	1.2579	46.22 ± 6.11*	132.41 ± 20.65
12	2-pentyl furan	C3777693	$C_9H_{14}O$	138.2	987.9	553.351	1.25478	91.88 ± 14.50	164.18 ± 25.87
13	methyl-5-hepten-2-one	C110930	$C_8H_{14}O$	126.2	985.1	547.199	1.17823	234.17 ± 27.40*	133.03 ± 1.73
14	(Z)-3-hexen-1-ol	C928961	$C_6H_{12}O$	100.2	864.4	342.987	1.23111	75.53 ± 4.22**	165.25 ± 7.26
15	Isopentyl propanoate	C105680	$C_8H_{16}O_2$	144.2	950.4	476.952	1.34298	212.26 ± 25.21**	44.21 ± 2.81
16	1-octen-3-ol	C3391864	$C_8H_{16}O$	128.2	977.6	531.117	1.15835	208.16 ± 10.10**	123.09 ± 7.06
17	Cyclohexanone	C108941	$C_6H_{10}O$	98.1	902.1	393.724	1.16128	148.29 ± 13.57**	51.04 ± 2.36
18	2-heptanone	C110430	$C_7H_{14}O$	114.2	884.2	367.983	1.26125	173.86 ± 27.96	139.87 ± 3.42

19	2(3H)-Furanone, dihydro-5-methyl-	C108292	$C_5H_8O_2$	100.1	930	439.767	1.13121	105.64 ± 3.65	90.54 ± 7.51	
20	Hexanal-M	C66251	$C_6H_{12}O$	100.2	785.3	258.849	1.26165	1102.06 ± 27.43	904.16 ± 66.61	Monomer
21	Hexanal-D	C66251	$C_6H_{12}O$	100.2	785	258.57	1.56392	664.94 ± 46.45	498.80 ± 91.18	Dimer
22	2-Hexanone	C591786	$C_6H_{12}O$	100.2	776.4	249.934	1.19238	139.70 ± 3.89	136.55 ± 4.23	
23	3-hydroxybutan-2-one-M	C513860	$C_4H_8O_2$	88.1	712.4	193.941	1.06266	404.69 ± 55.07	337.39 ± 10.22	Monomer
24	3-hydroxybutan-2-one-D	C513860	$C_4H_8O_2$	88.1	706.9	189.763	1.33218	129.08 ± 38.94	232.16 ± 50.43	Dimer
25	Pentanal-M	C110623	$C_5H_{10}O$	86.1	692	178.899	1.19112	211.73 ± 1.40**	166.26 ± 5.19	Monomer
26	Pentanal-D	C110623	$C_5H_{10}O$	86.1	689.6	177.227	1.39767	86.70 ± 1.96	94.54 ± 6.45	Dimer
27	2-Pentanone-M	C107879	$C_5H_{10}O$	86.1	682.7	173.884	1.12311	827.19 ± 31.57*	728.60 ± 1.40	Monomer
28	2-Pentanone-D	C107879	$C_5H_{10}O$	86.1	679.6	172.491	1.37374	1278.88 ± 73.31*	1538.14 ± 13.54	Dimer

29	2-methylbutanal-M	C96173	$C_5H_{10}O$	86.1	653.4	161.349	1.16593	246.40 ± 23.76*	145.38 ± 12.73	Monomer
30	3-methylbutanal-M	C590863	$C_5H_{10}O$	86.1	641	156.334	1.17979	322.49 ± 6.95**	197.44 ± 11.35	Monomer
31	2-methylbutanal-D	C96173	$C_5H_{10}O$	86.1	650.7	160.234	1.39515	221.74 ± 46.78	98.32 ± 15.62	Dimer
32	3-methylbutanal-D	C590863	$C_5H_{10}O$	86.1	643.8	157.449	1.40649	397.89 ± 88.19	167.95 ± 28.20	Dimer
33	2-butanone	C78933	C_4H_8O	72.1	566.2	129.135	1.24905	3037.56 ± 143.04	2912.77 ± 101.11	
34	2-Methyl-2-propenal	C78853	C_4H_6O	70.1	538.5	120.339	1.22236	243.83 ± 50.07	283.43 ± 28.29	
35	Ethanol	C64175	C_2H_6O	46.1	449.9	95.986	1.13709	1321.45 ± 85.58*	2410.11 ± 229.12	
36	Acetone	C67641	C_3H_6O	58.1	499.6	108.956	1.11427	1887.36 ± 466.69	3257.22 ± 394.03	Monomer
37	3-methylbutan-1-ol	C123513	$C_5H_{12}O$	88.1	726.8	205.34	1.24815	125.42 ± 5.06*	87.15 ± 9.90	
38	Methylpyrazine	C109080	$C_5H_6N_2$	94.1	800.4	273.097	1.08252	86.12 ± 13.00**	16.29 ± 1.33	

39	pent-1-en-3-ol	C616251	$C_5H_{10}O$	86.1	665.1	166.242	0.94147	181.94 ± 7.95	155.72 ± 12.86	Dimer
40	Butanal	C123728	C_4H_8O	72.1	535.8	119.502	1.28698	146.32 ± 9.44**	68.95 ± 1.71	
41	3-Methyl-2-butenal	C107868	C_5H_8O	84.1	771.2	244.819	1.09293	61.67 ± 14.32	38.72 ± 1.35	
42	n-Hexanol	C111273	$C_6H_{14}O$	102.2	863.7	342.127	1.32757	163.31 ± 15.21	117.72 ± 18.72	
43	pentan-1-ol	C71410	$C_5H_{12}O$	88.1	759	233.271	1.25491	70.91 ± 5.51	53.58 ± 5.96	
44	alpha-Phellandrene	C99832	$C_{10}H_{16}$	136.2	999.3	577.116	1.22509	91.21 ± 9.20	125.30 ± 19.99	
45	Ethyl Acetate	C141786	$C_4H_8O_2$	88.1	594.3	138.747	1.09594	44.32 ± 3.34	42.25 ± 1.71	

n = 3 per group indicates that nine fish were randomly selected from each group and pooled into three biological replicates for analysis. * indicates a significant difference ($P < 0.05$), ** indicates an extremely significant difference ($P < 0.01$). RI, Rt, Dt, and [RIP rel] denote the retention index, retention time, migration time, and normalized values, respectively.
